# Eviction of linker histone H1 by NAP-family histone chaperones enhances activated transcription

**DOI:** 10.1186/s13072-015-0022-8

**Published:** 2015-09-04

**Authors:** Qian Zhang, Holli A. Giebler, Marisa K. Isaacson, Jennifer K. Nyborg

**Affiliations:** Department of Biochemistry and Molecular Biology, Colorado State University, Fort Collins, CO 80523-1870 USA; Pace University, 1 Pace Plaza, New York, NY 10038 USA

**Keywords:** Linker histone, H1, Histone chaperone, SET/Taf1β, Chromatin, Transcription activation

## Abstract

**Background:**

In the Metazoan nucleus, core histones assemble the genomic DNA to form nucleosome arrays, which are further compacted into dense chromatin structures by the linker histone H1. The extraordinary density of chromatin creates an obstacle for accessing the genetic information. Regulation of chromatin dynamics is therefore critical to cellular homeostasis, and histone chaperones serve as prominent players in these processes. In the current study, we examined the role of specific histone chaperones in negotiating the inherently repressive chromatin structure during transcriptional activation.

**Results:**

Using a model promoter, we demonstrate that the human nucleosome assembly protein family members hNap1 and SET/Taf1β stimulate transcription in vitro during pre-initiation complex formation, prior to elongation. This stimulatory effect is dependent upon the presence of activators, p300, and Acetyl-CoA. We show that transcription from our chromatin template is strongly repressed by H1, and that both histone chaperones enhance RNA synthesis by overcoming H1-induced repression. Importantly, both hNap1 and SET/Taf1β directly bind H1, and function to enhance transcription by evicting the linker histone from chromatin reconstituted with H1. In vivo studies demonstrate that SET/Taf1β, but not hNap1, strongly stimulates activated transcription from the chromosomally-integrated model promoter, consistent with the observation that SET/Taf1β is nuclear, whereas hNap1 is primarily cytoplasmic. Together, these observations indicate that SET/Taf1β may serve as a critical regulator of H1 dynamics and gene activation in vivo.

**Conclusions:**

These studies uncover a novel function for SET that mechanistically couples transcriptional derepression with H1 dynamics. Furthermore, they underscore the significance of chaperone-dependent H1 displacement as an essential early step in the transition of a promoter from a dense chromatin state into one that is permissive to transcription factor binding and robust activation.

## Background

In the Metazoan nuclei, the extraordinary compaction of the genetic material is achieved through organization of the chromosomal DNA into extensively folded chromatin structures. Nucleosomes, the most basic unit of chromatin, are formed by wrapping 147 bp of DNA around two copies each of the core histones H2A, H2B, H3, and H4 [1]. The binding of the linker histone H1 further compacts the DNA into more condensed chromatin arrays [2]. The extensive compaction imparted by nucleosomes and the linker histones, in concert with other chromatin-associated proteins, creates a dense physical barrier that restricts access to the genetic information. As such, compacted chromatin structures are inherently incompatible with multiple nuclear processes, including gene expression. To enable access to promoters and regulatory elements of genes targeted for transcriptional activation, cells must possess mechanisms that actively locate these regions to locally relax the repressive chromatin fiber and expose specific DNA sequences.

The linker histone H1 plays a pivotal role in the formation and stabilization of extensively folded chromatin structures. In mammals there are at least 11 variants of H1. While structurally similar, with each subtype sharing a tripartite domain organization, the variants differ in their patterns of expression, chromosomal distribution and chromatin binding dynamics [3, 4]. The highly conserved central globular domain of the linker histones binds the nucleosome core particle at the entry/exit point of the DNA, further stabilizing the nucleosome and facilitating folding and compaction nucleosomal arrays into higher-ordered chromatin fibers [2, 4, 5]. Consistent with the repressive nature of H1, the association of the linker histones with chromatin inversely correlates with transcriptionally active gene regions. Recent genome-wide profiling studies revealed that the promoters of most transcriptionally active, or transcriptionally poised, genes are depleted of both nucleosomes and the linker histones [6–9]. However, unlike nucleosome-depleted regions that are generally localized to core promoters and enhancers, H1 displacement is considerably more extensive, extending significantly upstream and downstream of the transcription start site (TSS) [7–9]. These data further support the view that H1 binding is incompatible with gene expression, and that a critical early step in transcriptional activation is linker histone removal, followed by localized chromatin decompaction to accommodate the binding of the transcription machinery. Moreover, these observations indicate that specific cellular proteins and/or pathways must exist to facilitate H1 displacement from target gene regions primed for activation.

While many nuclear proteins modulate chromatin dynamics, the ATP-independent histone chaperones have established roles in numerous chromatin-related cellular processes, including gene expression [10, 11]. Members of the of nucleosome assembly protein (NAP) superfamily are amongst the best-characterized histone chaperones. The NAP proteins expressed throughout eukaryotes are structurally conserved and obligate dimers (or multimers), and their role in the regulation of chromatin dynamics is well documented [12]. Interestingly, despite the observation that their tertiary structure is conserved, differences exist in individual family members that likely support similar, yet distinct, chromatin-related functional properties [10, 11]. Members of the NAP superfamily bind the core histones with high affinity, and have been shown to promote nucleosome assembly [12], H2A/H2B dimer eviction and exchange [13–16], and acetylation-dependent nucleosome disassembly [17, 18]. Together, these activities support a prominent role for NAP-family proteins in regulated gene expression. In humans, there are at least eight NAP-superfamily members. These proteins differ with respect to their patterns of expression and subcellular distribution [19–21]. A subset of the NAP-family, NAP1L1-5, shares a greater degree of sequence homology, and thus the ability to heterodimerize given shared tissue expression and subcellular localization [19]. Of interest, all NAP1L1-5 proteins have been found in neurons, and a subset of these, hNAP1L2, L3, L5, exhibit restricted neuronal expression and dynamic changes in subcellular localization during differentiation [19]. The NAP-family members include hNAP1 (hNAP1L1) and hNAP2 (hNAP1L4) are ubiquitously expressed, as is the more distantly related NAP-family member, SET/Taf1 [also called SET, template activating factor-1 (Taf-1), INHAT, and IPP2A].

SET is predicted to share an overall structural homology with the NAP-superfamily member in mammals, despite the fact that it shares only 27 % amino acid sequence identity with hNAP1L1 [19]. The SET gene expresses two isoforms, SET/Taf1α and SET/Taf1β. Like the NAP proteins, SET is an obligate dimer, but does not heterodimerize with other NAP-family members, a finding consistent with observed pronounced differences in the dimerization helix [19, 22]. SET was originally discovered as a potent inhibitor of protein phosphatase 2A (PP2A) [23, 24]. Later, recurrent translocations involving SET and CAN (also called Nup214), a nucleoporin, were found to be associated with several distinct malignancies, including myeloid leukemias, colorectal, oral, and ovarian cancers [25–27]. The SET-CAN fusion protein dysregulates several potentially oncogenic nuclear processes, including aberrant retention of proteins targeted for export, stimulation of the *wnt* signaling pathway, and transcriptional activation of HOXA target genes [28–30]. Furthermore, misregulation of SET disrupts the catalytic activity of PP2A, which is linked to aberrant patterns of gene expression via effects on histone post-translational modifications [31], further linking SET to transcriptional regulation. Moreover, SET has recently been shown to play a role in chromosome segregation, suggesting that the SET/PP2A pathway is critical in the cell division cycle [32, 33]. Together, these diverse functions attributed to SET may be etiologically linked to malignant transformation associated with the deregulation of this important protein.

Although pleiotropic, SET has been shown to function in the regulated expression of numerous cellular genes. Its precise role in transcription, however, remains controversial. For example, several studies demonstrate that SET functions as a transcriptional activator via mechanisms that oppose the repressive effect of chromatin [31, 34–37], while other studies demonstrate SET functions as a repressor, primarily through inhibition of CBP/p300 acetylation activity [38–42]. Notably, the modulation of chromatin structure is a reoccurring theme in many studies that link SET and transcriptional regulation. Although the NAP-family proteins are well-characterized core histone chaperones, recent findings reveal that both NAP1 (hNAP1) and SET also interact with linker histone H1, and have been linked to chromatin decondensation through modulation of linker histone H1 dynamics [43–45]. However, the mechanisms by which these two human histone chaperones function in linker histone dynamics, and the potential outcome of the histone chaperone-H1 interaction on regulated gene expression, remain elusive.

In this report, we investigated the function of hNAP1 (hNAP1L1) and SET in a cell-free transcription system using a natural model promoter assembled into chromatin. We find that both chaperones enhance activated transcription, dependent upon the presence of activators, p300 and acetyl-CoA (AC-CoA). The stimulatory activity of hNAP1 and SET is significantly enhanced on chromatin templates assembled with H1. Importantly, both chaperones evict the linker histone from chromatin templates, providing a mechanistic foundation for their shared anti-repressive effects. Transfection of the histone chaperones into cells containing the integrated model promoter linked to a reporter plasmid reveals a potent transcriptional stimulatory function for SET, but not hNAP1. These data are consistent with the observation that SET is localized primarily in the nucleus. Further, transcriptional enhancement by SET is only observed with the integrated promoter, and under conditions of activated transcription. Together, these data uncover a novel transcription stimulatory function for SET that occurs early in transcriptional initiation: opposing H1 repression mediated through the targeted eviction of the linker histone from chromatin. Displacement of H1 is required for chromatin unfolding and the subsequent binding of the transcription machinery, creating a chromatin environment compatible with transcriptional activation.

## Results

### NAP1 proteins enhance activated transcription on chromatin templates during PIC assembly

**Fig. 1:**
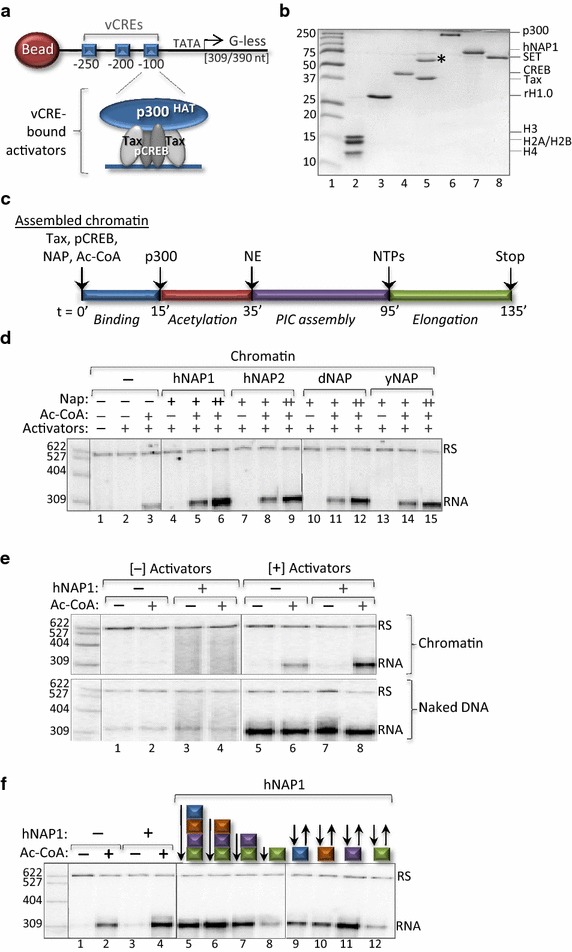
NAP proteins enhance activated transcription from an immobilized chromatin template. **a** Schematic depicting the model transcription template: the HTLV-1 promoter/G-less cassette. **b** Purified recombinant proteins. The indicated proteins used in the experiments described herein were separated by SDS-PAGE and visualized by Coomassie staining. Molecular weight size markers (kDa) (*lane 1*), *X. laevis* histone octamer (10 pmol) (*lane 2*), linker histone H1.0 (20 pmol) (*lane 3*), pCREB (Ser-133, 20 pmol) (*lane 4*), Tax (20 pmol) (*lane 5*); *asterisk* denotes GroEL that co-purifies with Tax, p300 (10 pmol) (*lane 6*), GST-hNap1 (20 pmol) (*lane 7*), and GST-SET/Taf1β (16 pmol) (*lane 8*). **c** Schematic representation of the in vitro transcription assay showing the individual, temporally-distinct steps. **d** NAP-family proteins enhance activated transcription from the chromatin-assembled promoter template. The repressive effect of chromatin was relieved by the addition of purified, recombinant activators (Tax, pCREB, and p300) and Ac-CoA. The indicated purified, recombinant histone chaperone (0.17 [+] or 0.67 μM [++]) was added in the presence of activators (Tax, pCREB and p300), and in the absence or presence of Ac-CoA (*lanes 4*–*15*). The amount of hNAP1 that gave the greatest enhancement (0.67 μM) was used in subsequent experiments. The transcript (*RNA*), recovery standard (*RS*), and size markers are shown. *Solid vertical lines* designate where the image (from the *same* gel) was spliced to remove irrelevant* lanes*, or portions of the image arranged to simplify presentation of the data. The experiment was performed at least three times, and a representative experiment is shown. For this, and subsequent in vitro transcription reactions, size markers derived from radiolabeled HpaII-digested pBR322 are shown on the *left* (bp). Prior to RNA isolation, a radiolabeled 622 bp recovery standard (*RS*) was added to each reaction to monitor transcript recovery. **e** hNAP1 stimulation of transcription requires activators, Ac-CoA, and chromatin. In vitro transcription assays were performed on chromatin-assembled (*upper panel*) or naked DNA (*lower panel*) templates, in the absence or presence of activators, as indicated. **f** hNAP1 enhances transcription during the PIC assembly step. In vitro transcription reactions, performed in the absence or presence of Ac-CoA and/or hNAP1 (added at the start of the reaction), is shown for reference (*lanes 1*–*4*). The effect of hNAP1 addition at the start of each individual step, denoted by color (see *panel* **b**) is shown (*lanes 5*–*8*). The effect of hNAP1, added at the start of the indicated step and removed from the reaction at the end of the indicated step (see “Results” for details), is shown (*lanes 9*–*12*). All reactions were performed in the presence of activators (Tax, pCREB, p300)

To investigate the role of NAP-family histone chaperones in transcription, we utilized a chromatin-based in vitro transcription system that we previously demonstrated to be highly responsive to activators, coactivators, and acetyl-CoA (Ac-CoA) [18, 46–48]. This model system is composed of a 588 bp (or 900 bp, see below) fragment carrying the natural HTLV-1 promoter linked to a G-less cassette immediately downstream of the TSS (Fig. 1a). The promoter fragment carries an upstream biotin linkage to enable immobilization on a streptavidin-coated magnetic bead. The HTLV-1 transcriptional control region carries three reiterated 21 bp enhancer elements, called viral cyclic AMP response elements (vCREs), located upstream of the TSS (Fig. 1a). The vCREs serve as binding sites for the cellular transcription factor CREB and the potent HTLV-1-encoded transcription factor Tax. DNA-bound Tax and ser-133 phosphorylated CREB (pCREB) together strongly recruit the coactivators CBP/p300 [48, 49] (Fig. 1a, b). CBP and p300 are ubiquitously expressed, highly homologous histone acetyltransferases (HATs) involved in the regulation of many classes of genes throughout metazoans [50]. Tax and pCREB recruitment of the coactivators is essential for potent, Ac-CoA-dependent transcription on chromatin templates in vitro [18, 47, 48].

Using our model cell-free transcription system we investigated whether NAP-family histone chaperones from different species functioned to influence activated transcription. Unless indicated otherwise, all transcription reactions were performed as follows: the immobilized chromatin-assembled model promoter templates [50 ng (0.004 µM)] were incubated in the presence of purified, recombinant activators [pCREB (0.03 µM), Tax (0.06 µM), p300 (0.02 µM)], the indicated histone chaperone (0.67 µM), and Ac-CoA (100 µM) (Fig. 1b). We tested purified, recombinant NAP from yeast, *Drosophila*, and the two ubiquitously expressed human NAP-family proteins, hNAP1 and hNAP2. A schematic depicting the temporal steps in the in vitro transcription reaction is shown in Fig. 1c. We first assembled chromatin on our immobilized model promoter by the method of salt dilution, and measured the effect of the NAP proteins on transcription (Fig. 1d). As expected, the chromatin-assembled promoter template was fully repressed in the absence of activators and Ac-CoA (lanes 1, 2). The addition of activators and Ac-CoA, however, produced an RNA transcript of the appropriate length (lane 3). Importantly, the addition of two amounts of each of the NAP proteins further increased the level of transcription in a dose- and Ac-CoA-dependent manner (lanes 4–15). We observed the most dramatic transcriptional enhancement in the presence of human NAP1. For this reason, and because the other components in our transcription system are of human origin, we selected hNAP1 for use in the subsequent studies presented herein. We next examined the enhancement effect of hNAP1 under a variety of transcription conditions (Fig. 1e). Consistent with a role for NAP-family proteins in modulating chromatin dynamics, we found that the transcription stimulatory effect of hNAP1 was only observed on chromatin templates, as hNAP1 addition had no effect on transcription reactions performed on naked DNA. Furthermore, transcriptional enhancement by hNAP1 required the presence of Tax, pCREB, p300, and Ac-CoA (Fig. 1e, and data not shown).

In the preceding experiments, NAP1 was added at the start of each reaction (*t* = 0′), and was present through termination (stop). To assess whether hNAP1 functioned optimally at a specific, temporally-distinct step in the transcription assay (see Fig. 1c), we tested the effect of hNAP1 when added at the start of each phase of the reaction. Figure 1f shows that hNAP1 enhanced transcription, except when added at the start of the elongation phase (lanes 5–8). This observation suggests that hNAP1 functions during assembly of the pre-initiation complex (PIC). To investigate this possibility further, we took advantage of our immobilized template system to specifically examine the effect of hNAP1 during each individual step in the reaction. hNAP1 was added at the start of each step, however, the supernatant containing hNAP1 was removed following magnetic isolation the transcription templates at the end of each step, and replaced with one prepared in parallel in the absence of hNAP1. Using this approach, the equilibrium binding conditions of the other reaction components remained essentially constant. Figure 1f shows that hNAP1 significantly enhanced transcription when present during PIC assembly (lane 11). It is unclear why NAP exhibited a repressive effect when added during the elongation phase of the reaction (lane 12). Collectively, these data demonstrate that hNAP1 is not a prototypical transcriptional activator, but rather functions on chromatin templates during the PIC assembly step to further stimulate activated transcription.

### Human NAP1 modulates the repressive function of histone H1 to enhance transcription

**Fig. 2:**
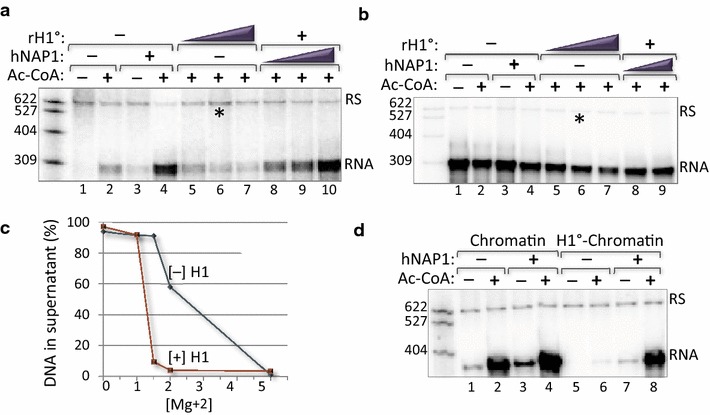
hNAP1 opposes the repressive function of linker histone H1 to enhance activated transcription in vitro. **a** hNAP1 reverses H1-induced transcriptional repression. In vitro transcription reactions were performed on the 588 bp HTLV-1/G-less cassette fragment as described (Fig. 1b), in the presence of activators and in the absence or presence of Ac-CoA, hNAP1, or rH1^0^. Reactions were supplemented with increasing amounts of rH1^0^ (0.03, 0.06, and 0.12 µM) at the start of the PIC assembly step (*lanes 5*–*7*). Reactions containing a constant amount of rH1^0^ (0.06 µM) were supplemented with increasing amounts of hNAP1 (0.17, 0.34 and 0.67 µM) (*lanes 8*–*10*). *Asterisk* denotes the rH1^0^ concentration used in reactions supplemented with varying amounts of histone chaperone. **b** Transcription from unassembled (*naked*) promoter templates is only modestly affected by H1 and hNAP1. The experiment was performed as described in *panel* **a**, except only two amounts of hNAP1 (0.34 and 0.67 µM) were added to reactions containing rH1^0^ (*lanes 8*, *9*). **c** rH1^0^-induced chromatin compaction. Chromatin was assembled on the 900 bp HTLV-1/G-less cassette fragment in the absence or presence of equimolar amounts of rH1.0 (H1:octamer), as indicated. The chromatin templates were incubated with increasing amounts of MgCl_2_, precipitated, and the percentage of DNA from chromatin prepared in the absence ([−H1], *blue*) or presence ([+H1], *red*) remaining in the supernatant following centrifugation was plotted. The *graph* shown is representative of three experiments. **d** hNAP1 relieves potent transcriptional repression induced by H1^0^-chromatin. Chromatin was assembled on the 900 bp HTLV-1/G-less cassette fragment in the absence or presence of equimolar rH1.0 (H1:octamer) (see “Methods”). hNAP1 (0.67 µM) was added to in vitro transcription reactions performed on chromatin (*lanes 1*–*4*) or H1-chromatin (*lanes 5*–*8*) templates, in the presence of activators, and in the absence or presence of Ac-CoA. Note that the 900 bp fragment produces an RNA transcript 390 nt in length

We next focused on the mechanism of hNAP1 transcriptional enhancement during PIC formation. Assembly of the PIC occurs following the addition of nuclear extract, as it supplies RNAP II, mediator, and the numerous additional factors essential for transcription initiation. Previous studies have reported that nuclear extracts also contain the linker histone H1, and that H1 is repressive to in vitro transcription [51–53]. Based on these observations, we hypothesized that hNAP1 enhances activated transcription via counteracting the repression induced by H1. To directly test this hypothesis, we added purified, recombinant H1^0^ (rH1^0^) at the beginning of the PIC assembly step. Figure 2a shows that exogenous H1^0^ repressed activated transcription (lanes 5–7). However, addition of increasing amounts of hNAP1 reversed rH1^0^ repression in a dose-dependent manner (lanes 8–10). Addition of rH1^0^ to reactions performed on naked DNA templates produced only a modest effect on transcription, indicating that H1-repression requires a chromatin context (Fig. 2b). Together, these data confirm that H1 is repressive to transcription in our in vitro system, and provide evidence that hNAP1 functions to enhance transcription by directly counteracting H1. We next examined the transcriptional stimulatory effect of hNAP1 on our 900 bp promoter fragment assembled into chromatin in the presence of equimolar amounts of rH1^0^ and histone octamer (H1^0^-chromatin). To verify the incorporation of the linker histone into our chromatin templates, we measured salt-dependent chromatin condensation [54]. Relative to chromatin arrays assembled with our promoter fragment in the absence of the linker histone, the rH1^0^-chromatin arrays precipitated at a lower Mg^2+^ concentration, indicating that H1 incorporation facilitated inter-array self-association and chromatin condensation (Fig. 2c). We next compared the transcriptional competences of rH1^0^-chromatin vs. chromatin to ascertain the functional consequences of H1 incorporation. Figure 2d shows that rH1^0^-chromatin was refractory to transcriptional activation (compare lanes 1, 2 and 5, 6). Importantly, the addition of hNAP1 reversed the potent repression induced by H1 incorporation into chromatin (compare lanes 6 and 8).

### The NAP-family protein, SET/Taf1β, similarly modulates the repressive function of histone H1 to enhance transcription

**Fig. 3:**
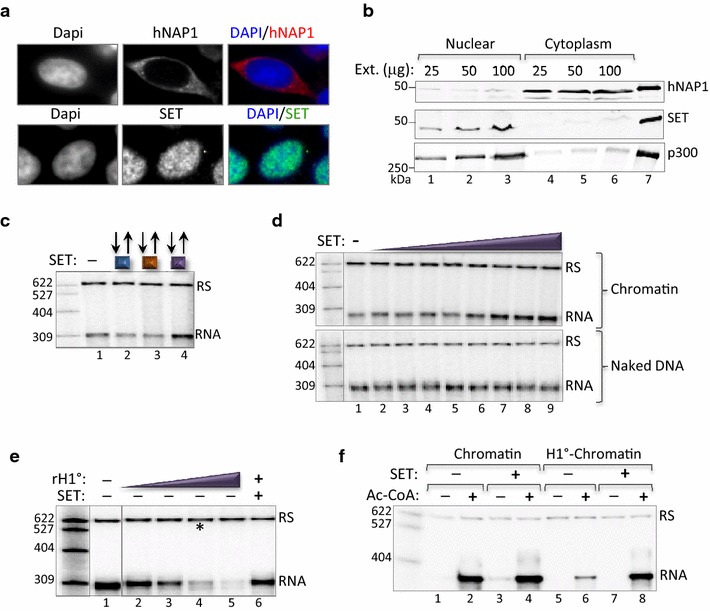
SET/Taf1β (SET), opposes the repressive function of H1 to enhance activated transcription in vitro. **a** Immunofluorescence labeling of hNAP1 (*red*, *upper panel*) and SET (*green*, *lower panel*) reveals distinct subcellular localization of the histone chaperones in HEK-293T cells. DAPI labeling of DNA is shown in *blue*. For single channel staining, *black* and *white* gave better resolution. For dual channel staining, *color images* were used to distinguish between the two antibodies. **b** Western blot analysis of hNAP1 and SET in nuclear and cytoplasmic extracts prepared from CEM T-cells. Immunostaining of nuclear p300 was performed to monitor efficiency of fractionation. Recombinant, purified hNAP1, SET, and p300 were analyzed in parallel as a positive control (*lane 7*). Recombinant hNAP1 migrates slightly slower due to the His_6_ purification tag. The position of the molecular weight size markers (kDa) is shown.** c** Like hNAP1, SET enhances activates transcription during the PIC assembly step. The in vitro transcription reaction was performed exactly as described for hNAP1 in Fig. 1e (*lanes 9*–*12*), in the presence of activators and SET (0.67 µM). **d** Dose-dependent enhancement of transcription by SET requires chromatin. In vitro transcription assay performed with increasing amounts of SET (0.03, 0.06, 0.12, 0.2, 0.27, 0.33 and 0.67 µM) added during PIC assembly produced a dose-dependent enhancement of transcription from chromatin templates (*upper panel*), but not from naked DNA (*lower panel*). The experiment was performed in the presence of activators and Ac-CoA.** e** SET reverses rH1^0^-induced transcriptional repression. The in vitro transcription experiment was performed as described in Fig. 2a, with increasing amounts of rH1^0^ (0.015, 0.03, 0.06, and 0.12 μM) (*lanes 2*–*5*), or a constant amount of rH1^0^ (0.06 μM*) and SET (0.34 μM) (*lane 6*), in the presence of activators and Ac-CoA.** f** SET reverses transcriptional repression induced by H1^0^-chromatin. In vitro transcription reactions were performed on the 900 bp HTLV-1/G-less cassette fragment, exactly as described for Fig. 2d, using SET (0.67 µM) in place of hNAP1. Reactions were performed in the presence of activators, and the absence or presence of SET and/or Ac-CoA, as indicated

The data presented above provide strong evidence that hNAP1 enhances transcription through opposing H1 repression. The physiological significance of these observations is uncertain, however, as many NAP-family members have been reported to reside primarily, although not exclusively, in the cytoplasm [21, 55, 56]. SET/Taf1β (SET) shares structural similarity to NAP1, however, it has been shown to reside primarily in the nucleus [45, 57]. Further, SET has been implicated in chromatin decondensation, H1 dynamics, transcriptional regulation, and PIC formation [34, 35, 38, 39, 45, 57–60]. Based on these observations, we turned our focus to SET as a potential candidate histone chaperone that may also function to enhance transcription by opposing H1-repression. We first analyzed the sub-cellular localization of hNAP1 and SET/Taf1β by immunofluorescence and cell fractionation. Figure 3a, b reveal that, in the cell lines examined, hNAP1 is predominately cytoplasmic, whereas SET is predominately nuclear. However, it should be noted that the NAP-family proteins carry both nuclear import and export signals. As such, NAP may perform chromatin-related nuclear activities either transiently, under certain cellular conditions (e.g., during development or a specific phase of the cell cycle), and/or function at relatively low concentrations. We next examined SET function during the individual steps in the transcription reaction. Similar to hNAP1 (see Fig. 1e), SET enhanced activated transcription during the PIC assembly step of initiation (Fig. 3c). Increasing concentrations of SET produced a dose-dependent stimulation of transcription from the chromatin-assembled templates, but had no effect on transcription from naked DNA (Fig. 3d). Importantly, SET also reversed the repression induced by the addition of rH1^0^ to the transcription reactions, and countered the potent repression induced by rH1^0^-chromatin (Fig. 3e, f). Taken together, these observations indicate that both hNAP1 and SET likely function in a mechanistically similar way to enhance transcription by counteracting H1 repression.

### SET/Taf1β and hNAP1 directly bind H1 and evict the linker histone from chromatin

**Fig. 4:**
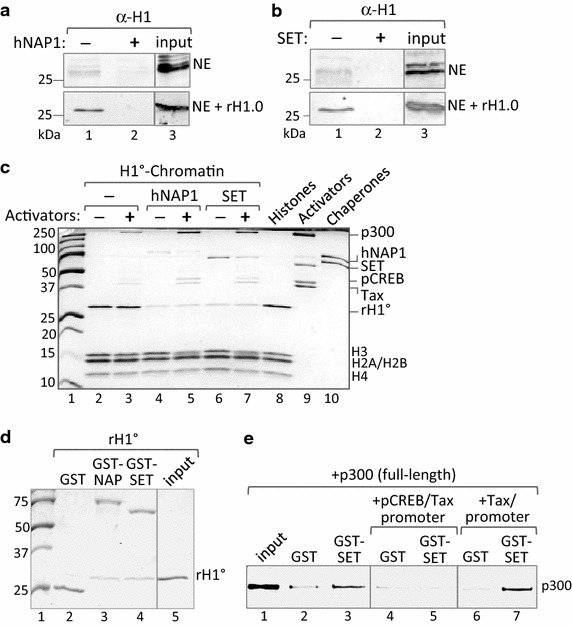
hNAP1 and SET evict H1 from chromatin. **a**, **b** hNAP1 and SET remove endogenous H1 and rH1^0^ from chromatin templates. Immobilized chromatin templates (0.33 ng/µl) were incubated with CEM NE (*upper panel*) (~0.55 µg/µl), or NE supplemented with rH1^0^ (*lower panel*) (0.067 µM; ~fourfold excess rH1^0^ relative to octamer) in the absence or presence of hNAP1 (*panel* **a**) or SET (*panel* **b**) (0.67 µM each). The binding assays were designed to replicate the conditions of the in vitro transcription reactions shown in Fig. 2 (hNAP1) and 3 (SET) (i.e., transcription in the presence of NE, or NE supplemented with exogenous rH1^0^). For each* panel*, input NE (15 %) and rH1^0^ (100 %) are shown (*lane 3*). Note that input H1 appears similar due to saturating amounts of protein. H1 binding was assessed by Western blot using an antibody against rH1^0^. **c** hNAP1 and SET remove H1 incorporated into chromatin. The 900 bp immobilized promoter fragment was assembled into H1^0^-chromatin, followed by incubation with activators, Ac-CoA, and histone chaperones, as indicated. For reference, input amounts (100 %) of histones (octamer, rH1^0^), activators (Tax, CREB, p300), and chaperones were analyzed in parallel (*lanes 7*–*9*). Note that in the reactions containing activators, p300, Ac-CoA, and histone chaperones, p300 acetylation caused diffuse migration of the H3 band (*lanes 4*, *6*). Under the conditions of the experiment, only histone H1 was evicted from the promoter template. Reactions were analyzed by SDS-PAGE followed by Coomassie staining. Molecular weight size markers (kDa) are shown (*lane 1*). **d** hNAP1 and SET directly bind rH1^0^ as demonstrated by GST pull-down assay. Input rH1^0^ (50 %) (*lane 5*) and molecular weight size markers (kDa) (*lane 1*) are shown. Binding reactions were analyzed by SDS-PAGE followed by Coomassie staining. **e** SET binding to full-length p300 is abolished in the presence of enhancer-bound Tax/pCREB complex. GST pull-down assays were performed to measure the binding of p300 to SET in the absence or presence of HTLV-1 promoter-bound activators (Tax/pCREB). Full-length p300 bound to GST-SET alone (*lanes 2*, *3*), but was disrupted in the presence of the Tax/pCREB-bound promoter DNA (which serves as a high affinity binding site for p300) (*lanes 4*, *5*). The p300/SET interaction was unaffected under the same conditions, but in the absence of pCREB (*lanes 6*, *7*). Input p300 (100 %) is shown (*lane 1*). Binding was analyzed by Western blot using an antibody against p300

We next investigated the mechanism of histone chaperone-mediated anti-repression. Using the immobilized template assay, and binding conditions designed to precisely recapitulate the transcription reactions shown in Figs. 2a and 3e, we found that H1 supplied by the nuclear extract, or rH1^0^ added exogenously to the reaction, directly bound the chromatin-assembled promoter template (Fig. 4a, b, lane 1). Interestingly, based on the input amounts, the nuclear extract contributes significantly more total H1 to the binding reaction than exogenously added rH1^0^ (Panels a, b, lanes 3). However, the rH1^0^ appears to bind more efficiently to the templates, suggesting that a significant percentage of the endogenous H1 may be modified or in complex with other components in the reaction, and thus is unavailable for binding. Of note, the addition of hNAP1 and SET to the binding reactions reduced H1 binding to the template (Fig. 4a, b, lane 2). These data confirm that H1 is present in the nuclear extracts and that it binds to the transcription templates. Further, H1 binding likely imparts a moderately repressive effect on transcription that is relieved, via displacement, by the histone chaperones. We next measured the effect of hNAP1 and SET on H1 incorporated into chromatin. Following H1-chromatin assembly, the immobilized templates were incubated in the absence or presence of activators, p300, hNAP1 and SET. Figure 4c shows that both chaperones significantly reduced the amount of H1 incorporated into chromatin, and that H1 displacement was independent of activators and histone acetylation by Ac-CoA (lanes 3–6). Analysis of the supernatant fraction confirmed the presence of evicted H1, but not the core histones (data not shown). Of note, the chaperone-mediated release of rH1^0^ from the templates correlated with increased transcription factor/coactivator binding, an observation consistent with chromatin relaxation following H1 release. To further decipher the mechanism of chaperone-dependent H1 displacement, we performed GST pull-down assays and confirmed that both chaperones directly bind rH1^0^ (Fig. 4d) [43–45]. Interestingly, these observations support a pathway for chaperone-mediated H1 eviction; however, the mechanism of chaperone recruitment to H1-containing promoters remains elusive. Previous reports, together with our unpublished studies, have shown that the NAP-family proteins directly bind to CBP/p300 [61, 62], suggesting a potential mechanism of coactivator-mediated recruitment to promoters targeted for activation. However, as shown in Fig. 4d, we did not observe enhanced association of either hNAP1 or SET in binding reactions containing p300. In general, we found that chaperone binding to the immobilized template varies between experiments, but is generally low or undetectable. From these observations, we hypothesized that a chaperone–coactivator complex exists in solution, and that the chaperone is released upon coactivator recruitment to the promoter. To test this hypothesis, we examined full-length p300 binding to GST-SET, in the absence or presence of the enhancer-bound Tax/pCREB complex. GST pull-down assays confirmed complex formation between p300 and SET in solution (Fig. 4e, lanes 2, 3). However, when the p300/SET interaction was analyzed in the presence of the Tax/pCREB/vCRE DNA complex, which binds p300 with high affinity, the p300/SET interaction was abolished (lanes 4, 5). In contrast, when the p300/SET complex was similarly challenged with Tax/vCRE DNA, which does not bind p300, the p300/SET interaction was unaffected (lanes 5, 6). These data support a model in which the histone chaperone is initially recruited to the promoter via direct interaction with CBP/p300, followed by chaperone release from the coactivator to facilitate H1 eviction. Together, these findings provide a mechanistic foundation for the anti-repression activity of the histone chaperones.

### SET/Taf1β enhances activator-dependent transcription in vivo

**Fig. 5:**
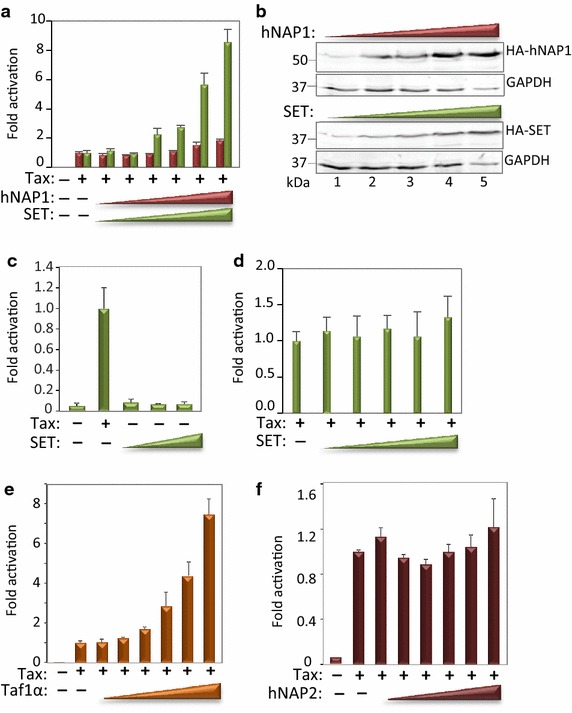
SET enhances activated transcription in vivo. **a** SET, but not hNAP1, enhanced activated transcription from the chromosomally-integrated model promoter. CHOK-Luc cells, carrying multiple integrated copies of the HTLV-1 promoter linked to luciferase [63] were transiently transfected with an expression plasmid for Tax alone (10 ng) or co-transfected with increasing amounts (25, 50, 100, 200, 400, 500 ng) of HA-hNAP1 or HA-SET expression plasmids. The signal obtained in the presence of Tax alone was normalized to 1, and fold activation by the histone chaperones, relative to Tax alone, is reported. The experiment was performed in triplicate, and is representative of three biological replicates. **b** Expression of HA-hNAP1 (*upper panel*) and HA-SET (*lower panel*) following transfection. CHOK-Luc cells were transfected as described in *panel* **a**, and lysates analyzed by immunoblot using an anti-HA antibody. Immunoblot analysis of GAPDH was performed in parallel as a loading control. **c** SET enhancement of transcription requires co-transfection of Tax. CHOK-Luc cells were transiently transfected with an expression plasmid for Tax (10 ng) or the expression plasmid for SET (200, 400, 500 ng), as indicated. **d** The transiently transfected HTLV-1 promoter/luciferase reporter plasmid is refractory to enhancement by co-transfected SET. Jurkat T-cells were transiently co-transfected with the HTLV-1/Luc reporter plasmid (100 ng) [87], and expression plasmids for Tax and SET, as described in *panel* **a**. **e** SET/Taf1α, an isoform of SET/Taf1β, enhanced activated transcription from the integrated model promoter. The experiment was performed as described in *panel* **a**, with co-transfected Taf1α in place of SET. **f** Co-transfection of hNAP2 had a negligible effect on activated transcription from the integrated model promoter. The experiment was performed as described in *panel* **a**

We next asked whether the NAP family histone chaperones functioned to enhance activated transcription in vivo. To recapitulate our in vitro model system, we utilized a cell line that carries the chromosomally-integrated HTLV-1 promoter linked to luciferase [63]. Of note, we previously demonstrated that Tax-induced transcriptional activation in this cell line correlated with eviction of H1 from the HTLV-1 promoter [64]. To test whether expression of the chaperones enhanced activated transcription, we co-transfected an expression plasmid for Tax, in the absence or presence of increasing amounts of the expression plasmids for SET or hNAP1. As expected, Tax dramatically stimulated expression from the integrated HTLV-1 promoter (150-fold) (Fig. 5a). Importantly, co-transfection of SET, but not hNAP1, significantly enhanced Tax transactivation in a dose-dependent manner. Expression of the chaperones was confirmed by immunoblot analysis (Fig. 5b). Consistent with our in vitro data, the stimulatory effect of SET required conditions of activated transcription, as SET had no effect in the absence of Tax (Fig. 5c). Moreover, Tax-activated transcription was refractory to stimulation by SET when assayed on a transiently transfected HTLV-1 promoter reporter plasmid (Fig. 5d), consistent with a requirement for properly assembled H1-chromatin [65]. To investigate the potential stimulatory effect of other NAP-superfamily histone chaperones on transcriptional activation, we also tested Taf1α, an isoform of SET/Taf1β, and hNAP2 (hNAP1L4). Like SET, transfection of Taf1α also enhanced Tax transactivation (Fig. 5e). Similarly, like hNAP1, the related hNAP2 had no effect on transcription from the integrated model promoter (Fig. 5f). These findings consistent with the respective subcellular localization of the histone chaperones, as well as the in vitro binding and functional data, and together support a role for SET in transcriptional activation via modulation of H1 dynamics.

## Discussion

The studies presented herein provide the first report to directly link the histone chaperones NAP1 (hNAP1L1) and SET/Taf1β with histone H1 displacement and transcriptional activation. Using a powerful, chromatin-based in vitro system, we demonstrate that both human NAP1 and SET enhance transcription from an immobilized native promoter template. Transcriptional enhancement is dependent upon a chromatin environment, and the presence of activators (Tax and pCREB), p300, and Ac-CoA. The underlying requirement for transcriptionally permissive conditions indicates that the histone chaperones exert their stimulatory effect in a manner distinct from conventional DNA-binding transcription factors. To explore the mechanism of transcriptional enhancement, we examined histone chaperone function during each temporally-distinct step of the initiation reaction. We initially found that both hNAP1 and SET exert their stimulatory activity during PIC assembly, immediately following nuclear extract addition. Based on the unconventional activation properties of the histone chaperones, we considered the possibility that they functioned to enhance transcription via displacement of a repressor, thus facilitating PIC formation. Previous studies found that nuclear extracts contain the repressive linker histone H1, and that H1 is potently repressive to transcription [51–53]. Since both chaperones bind to the linker histones, we reasoned that hNAP1 and SET might function as anti-repressors; enhancing transcription by opposing H1-induced repression. Using the immobilized template assay, we find that endogenous and exogenous H1 bind to the chromatin-assembled model promoter template, and that exogenous H1 potently represses transcription. The level of transcription stimulation by the chaperones directly correlates with the amount of H1 present in the assay. While both chaperones stimulated transcription on chromatin templates without exogenously added H1 (likely through displacement of H1 in the nuclear extract), their overall stimulatory effect is appreciably enhanced in the presence of exogenously added H1. Significantly, restoration of high-level activated transcription by the histone chaperones directly correlates with reduced H1 binding to the template, increased activator and coactivator binding, and histone H3 acetylation; all conditions essential for strong transcription. Our findings are consistent with a previous study showing that both NAP1 and SET enhance activated transcription on chromatin templates in vitro, at a step prior to elongation [35]. However, the mechanism of transcriptional enhancement—specifically the involvement of H1—was not identified.

We find that in vitro, NAP1 and SET function in a mechanistically indistinguishable manner to derepress transcription from our model promoter. In vivo, however, SET, but not hNAP1, significantly enhances transcription from the stably integrated HTLV-1 promoter/reporter construct. This observation is consistent with the predominant cytoplasm localization of NAP1 and nuclear localization of SET. Because H1 is an abundant nuclear protein, it is improbable that hNAP1 would have a general role in modulating H1 dynamics in the cell lines examined. It should be noted, however, that NAP1 carries both nuclear import and export signals [12, 66]. Furthermore, NAP1 has been shown to bind both core and linker histones, and has been implicated in numerous transcription-related processes [10, 11]. Based on these observations, the nuclear distribution of hNAP1, and thus its role in H1 dynamics, may be influenced by post-translational modification (phosphorylation or glutamylation), host cell and/or stage of differentiation, or the presence of a compatible dimerization partner. We also found that the anti-repressive activity of SET in vivo is dependent upon Tax expression, a result that corroborates our in vitro observations. Furthermore, SET enhanced transcription from the chromosomally integrated model promoter/reporter construct, but had no effect when the same reporter construct was transiently transfected into the cells. This finding underscores the requirement for a native chromatin environment for SET transcriptional enhancement function, and further implicates H1 as a major target of SET. Moreover, we previously detected significant eviction of H1 from the integrated HTLV-1 promoter region in this same cell line [64]. H1 loss was dependent upon Tax expression, and correlated with activator and coactivator binding, RNA polymerase II recruitment, and strong transcriptional activation. Our previous study demonstrating activator-dependent H1 eviction, together with the studies presented herein, are consistent with a model of chaperone-mediated anti-repression via eviction of H1. Displacement of the linker histone is likely an early event required for relaxation of the chromatin fiber, followed by nucleosome mobilization and PIC formation. Although the role of the linker histones in gene regulation has been controversial [4, 67], recent genome-wide profiling studies demonstrate significantly reduced H1 association at active promoter regions [7–9]. These observations are consistent with a model of activated transcription in which H1 displacement is an early event in transcriptional initiation. The strong correlation between our in vivo and in vitro data support a role for SET as an H1 chaperone responsible for transcriptional derepression mediated through the targeted displacement of the repressive linker histone.

These studies begin to address the mechanism by which SET is recruited to the promoters of genes targeted for H1 displacement. In a previously published study examining heat shock genes in *Drosophila* polytene chromosomes, SET was shown to dramatically redistribute to transcriptionally active gene regions following heat shock [31], however, the mechanism/s that facilitated targeted relocalization of SET to the heat shock loci remained elusive. As described herein, H1 eviction directly correlates with the binding of the activators and p300 to the model promoter. These observations infer a causal relationship between activator/coactivator binding and H1 dissociation. Further, they implicate a role for the transcription factors and/or p300 in the delivery of SET to the viral enhancer elements for H1 dissociation. In support of this idea, previous studies indicate that SET may directly interact with CBP/p300 and specific transcription factors [68]. These studies offer precedence for a mechanism of direct recruitment of SET to enhancer regions prior to transcriptional activation. While we did not detect stable association of SET with promoter-bound p300 in vitro, we have confirmed an interaction between the two proteins in solution. Moreover, we find that the SET/p300 complex is destabilized upon p300 binding to the HTLV-1 enhancer. These data provide preliminary support for a model of targeted recruitment of SET via coactivator binding and subsequent release at the promoter to facilitate local eviction of H1. Of note, we observe the binding of activators and p300 to H1-chromatin, albeit at a reduced level relative to chromatin depleted of H1 (see Fig. 4c). These observations provide support for a model in which activators and coactivators associate with the promoters of genes targeted for activation prior to chromatin decondensation, and thus provide a mechanism for the precise delivery of SET, followed by H1 displacement. As such, H1 displacement may be an early event in initiation, but one that occurs subsequent to the binding of specific transcription factors and/or coactivators that serve to elicit the cascade of events that culminate in transcriptional activation.

## Conclusions

We biochemically characterized a novel anti-repressor function of members of the NAP superfamily; the opposition of H1 transcriptional repression mediated by the physical displacement of H1 from chromatin. Eviction of the linker histone by the chaperones SET and hNAP1 promotes chromatin decompaction and the creation of a relaxed chromatin environment permissive to PIC formation and transcriptional initiation. Although NAP1 and SET function indistinguishably in vitro to displace H1 from chromatin and enhance activated transcription, the abundant nuclear localization of SET, together with strong enhancement of activated transcription in vivo, collectively support a biologically-relevant role for SET as a novel transcriptional anti-repressor that functions via modulation of H1 dynamics. Of note, previous in vivo studies correlated SET overexpression with chromosomal decondensation and enhanced H1 exchange [45, 58, 69, 70], whereas silencing of SET correlated with abnormally condensed chromosomes [45]. Our novel biochemical studies integrate the numerous previous studies on SET, and identify a direct link between SET and H1 mobilization. Together, our findings support a mechanism for achieving chromatin decompaction in vivo, and point to a physiologically-relevant role for SET in targeting specific genes for transcriptional activation.

## Methods

### Plasmid constructs, protein expression, purification, nuclear extract

The mammalian reporter plasmid, HTLV-Luc [71], and expression plasmids, pSG-Tax [72], pcDNA-HA-hNAP1 and pCMV-myc-hNAP2 [73] have been described previously. The HA-SET/Taf1 (pcDNA-HA-Taf1α/β) mammalian expression plasmid was subcloned from the bacterial expression plasmid [74]. Each of the following recombinant proteins used in this study were expressed and purified as described in their accompanying references, or using established protocols: Tax-His_6_ [75], ser-133 phosphorylated CREB (pCREB) [76, 77], GST-hNAP1 and His_6_-hNAP1 [73], His_6_-dNAP [78], His_6_-GST-SET/Taf1β, and linker histone rH1.0 [79, 80]. Purified, recombinant *X. laevis* core histones were assembled into octamer as previously described [46, 81].

### Cell culture, nuclear extracts, luciferase assays

CHOK1-Luc hamster ovary cells (gift from Dr. Kuan-Teh Jeang) [63], HEK-293T, and HeLa cells were cultured in DMEM (Sigma) supplemented with 10 % fetal bovine serum (FBS), 2 mM l-glutamine, and penicillin–streptomycin. CHOK1-Luc cells were supplemented with 500 μg/ml G418 to maintain the integrated HTLV-1/Luc reported construct. CEM T-cells and Jurkat T-cells were maintained in IMDM (Gibco) supplemented with 10 % FBS, 2 mM l-glutamine, and penicillin–streptomycin. HeLa S3 cells, grown in suspension for nuclear extract preparation, were cultured in IMDM supplemented with 2 % FBS, 2 mM l-glutamine, and penicillin–streptomycin. Nuclear extracts for transcription assays were prepared from HeLa S3 or CEM cells as previously described [82]. Both extracts gave the same results in the transcription assays. Luciferase assays using CHOK1-Luc cells [63] or Jurkat T-cells were performed as previously described [64]. Briefly, 1 × 10^5^ cells were seeded into a 24-well plate and transfected with the indicated plasmids and a constant amount of DNA (1 μg) using Lipofectamine reagent (Invitrogen, Carlsbad, CA, USA) according to the manufacturer’s protocol. After 48 h the cells were harvested and lysed, and luciferase activity was measured using a dual-luciferase reporter assay system (Promega). Luciferase activity was detected using a Turner Designs luminometer *(*TD-20/20). To control for transfection efficiency, cells were co-transfected with *Renilla*, and firefly luciferase activity was normalized to *Renilla* activity (pRL-TK, Promega, Madison, WI, USA). Because the luciferase signal is very low in the absence of transfected Tax, we set the signal obtained in the presence of Tax to 1, and normalized all other samples relative to the signal obtained with Tax alone. The transient transfection assays were performed in triplicate with at least three biological replicates.

### Chromatin assembly

Immobilized templates (588 or 900 bp) carrying the HTLV-1 promoter linked to a G-less cassette (HTLV-1/G-less) were generated by PCR using an upstream biotinylated primer. The biotinylated promoter fragments were immobilized on streptavidin-coated magnetic Dynabeads (Invitrogen) according to the manufacturer’s instruction. The immobilized DNA templates were assembled into chromatin (0.8:1 histone octamer/DNA ratio [wt/wt]) by salt deposition as described previously [48]. Briefly, bead-bound DNA fragment and histone octamer were mixed at 4 °C at 1300 rpm in 10 mM Tris, pH 8.0 mM EDTA (TE) containing 1 M NaCl and 0.1 % Chaps. The NaCl concentration was diluted stepwise to 0.8, 0.6, 0.4, 0.2, 0.1 M, with a 30 min. agitation per step. All transcription reactions, except those with H1^0^-chromatin, were performed with the 588 bp promoter fragment, which gives a transcript 310 nt in length. For H1^0^-chromatin assembly, equimolar amounts of rH1^0^ and histone octamer were added at the beginning of chromatin assembly using the 900 bp fragment. The longer fragment gives a transcript 390 nt in length. The longer fragment was initially used to promote higher order H1-chromatin structure formation, however we obtained essentially identical transcription results with either the 588 or 900 bp fragments assembled into H1-chromatin. The assembled templates were stored at 4 °C in storage buffer (1 mM EDTA, 10 mM NaCl, 0.1 % NP40, 0.5 % Chaps, 20 % glycerol, 2 mM DTT) at a DNA concentration of 100 ng/μl, and used within 1 week.

### In vitro transcription assay

The immobilized chromatin-assembled promoter templates (50 ng; 0.004 μM) were used in transcription assays as previously described [17, 18]. Briefly, the bead-bound promoter fragments were incubated with pCREB, Tax, and Ac-CoA (as indicated in each reaction) for 15′ at 30 °C with shaking (1300 rpm) followed by the addition of p300 for 20′ (see Fig. 1c). Nuclear extract (~15 μg) was added to each reaction and incubated for an additional 60′ in a final volume of 30 μl in 0.5× TM buffer (25 mM Tris, pH 7.9, 50 mM KCl, 6.25 mM MgCl_2_, 10 % glycerol, 0.5 mM EDTA, 0.5 mM DTT). RNA synthesis was initiated by addition of 224 μM ATP, 224 μM CTP, and 0.75 μM [α-^32^P] UTP (3000 Ci/mmol), and the elongation reaction incubated for an additional 40 min. Reactions were stopped, RNA was isolated following digestion with Proteinase K, and transcripts were separated by denaturing PAGE and analyzed using ImageQuant^®^ software. Molecular weight markers (radiolabeled HpaII-digested pBR322) were used to estimate the sizes of the RNA products. A labeled 622-bp DNA fragment was added to each reaction mixture as a recovery standard. Unless otherwise indicated, constant amounts of the following purified, recombinant proteins were used: pCREB (0.03 μM), Tax (0.06 μM), p300 (0.02 μM), hNAP1 (0.67 μM), SET/Taf1β (0.67 μM) and Ac-CoA (100 μM). Experiments in which the chaperone was added and removed at a specific step were performed as follows: after the indicated incubation, the supernatant was removed by magnetic isolation of the immobilized transcription templates, and replaced with supernatant generated in parallel, but lacking the histone chaperone. Using this approach, the overall steady-state concentrations of each reaction component remained constant. All transcription experiments were performed at least three times, with each replicate giving essentially identical results.

### Immobilized template assay

Binding reactions were prepared as described for the in vitro transcription assays, except the reactions were increased 20-fold (1 μg of DNA) to enable visualization of template-bound proteins by Coomassie-stained gels. Unless otherwise indicated, reactions contained purified, recombinant pCREB (0.03 μM), Tax (0.06 μM), p300 (0.02 μM), hNAP1 or SET/Taf1β (0.67 μM), and Ac-CoA (100 μM) in 0.5× TM buffer. At the completion of the binding reaction, the bead-bound templates were isolated by magnetic separation, and washed twice with 0.5× TM buffer. Template-bound proteins were separated by 15 % SDS-PAGE and analyzed by Western blot (Fig. 4a, b) or Coomassie blue staining (Fig. 4c).

### Analysis of H1-chromatin

Magnesium-dependent chromatin condensation assays were performed as previously described [83, 84]. Chromatin and H1-chromatin was assembled as described above, except using the method of salt dialysis, as described [85]. Following dialysis, arrays were incubated in the presence of the indicated concentration of MgCl_2_ and subjected to centrifugation for 10 min at 16,100*g*. DNA from both the pellet and supernatant fractions was purified by phenol/chloroform extraction and ethanol precipitation, and analyzed by 1 % agarose gel electrophoresis followed by ethidium bromide staining. DNA from each fraction was quantified using ImageQuant^®^ software and plotted as percentage of total DNA in each reaction.

### GST pull-down assay

GST pull-down assays were performed as described [86]. Briefly, purified GST alone, or GST-fused histone chaperone (25 pmol) were bound to 20 μl glutathione agarose beads in binding buffer (50 mM Tris–HCl, pH 7.9, 150 mM KCl, 12.5 mM MgCl_2_, 1 mM EDTA, 20 % glycerol, 0.5 % NP40, 0.1 % Chaps, and 100 ng/μl BSA) for 1 h at 4 °C, followed by incubation with an equal concentration of rH1.0 for an additional 16 h at 4 °C. The beads were washed extensively, and GST-bound proteins analyzed by 15 % SDS-PAGE and Coomassie blue staining (Fig. 4d) or western blot (Fig. 4e). For the GST pull-down reactions using full-length p300, the binding reactions contained GST alone or GST-SET (20 pmol) and full-length p300 (5 pmol). The binding reactions were performed in the absence or presence of activator/DNA complex composed of the 588 bp HTLV-1 promoter DNA fragment (2.2 pmol; 6.6 pmol binding sites) and either Tax alone (which does not bind DNA in the absence of CREB), or Tax/pCREB (30 pmol each, 15 pmol dimers). Binding reactions were performed as described above in the presence of 0.5× TM buffer supplemented with 0.1 % NP40 and 500 ng/μl BSA.

### Antibodies, western blot and immunofluorescence labeling

Western blot and immunofluorescence labeling were performed according to the manufacturer’s instructions. Anti-hNAP1 (1:2000, Abcam #21630), anti-SET/Taf1β (1:2000, Abcam #1183), anti-p300 (1:300, Santa Cruz Biotechnology #584), anti-H1 (1:300, Santa Cruz Biotechnology #8030), anti-HA (Santa Cruz Biotechnology #SC-805), and anti-GAPDH (Santa Cruz Biotechnology #SC-137179) were used for western blot analysis. Immunofluorescence labeling in HEK-293T cells was performed using anti-hNAP1 (1:150, Abnova #H00004673) and anti-SET/Taf1β (1:200, Abcam #1183) primary antibodies and Alexa Fluor^®^ 488 goat anti-rabbit IgG (H+L) (1:200, Invitrogen #A11008) and Alexa Fluor^®^ 594 goat anti-mouse IgG (H+L) (1:200, Invitrogen #A11005) secondary antibodies. An Olympus IX81 spinning disk confocal microscope with 60×/1.42 NA objective and a Photometrics Cascade II CCD camera with SlideBook (Intelligent Imaging Innovations) software were used for fluorescence images. Metamorph software was used to process images.

### Image processing

Solid vertical lines on some gels indicate where the image (from the *same* gel) was spliced to either remove irrelevant lanes or enhance presentation of the data. Minimal and uniform brightness/contrast was applied to some gel images.

## Abbreviations

NAP: nucleosome assembly protein
SET: also called SET/Taf1β, template activating factor-1 (Taf-1), INHAT, and IPP2A
TSS: transcription start site
CREB: cAMP response element binding protein
CBP: CREB binding protein
pCREB: phosphorylated CREB
Ac-CoA: acetyl coenzyme A
HTLV-1: human T cell leukemia virus, type 1
PIC: preinitiation complex assembly
vCRE: viral cAMP response element
GST: glutathione-S-transferase
